# A Constitutively Mannose-Sensitive Agglutinating *Salmonella enterica* subsp. *enterica* Serovar Typhimurium Strain, Carrying a Transposon in the Fimbrial Usher Gene *stbC*, Exhibits Multidrug Resistance and Flagellated Phenotypes

**DOI:** 10.1100/2012/280264

**Published:** 2012-04-30

**Authors:** Kuan-Hsun Wu, Ke-Chuan Wang, Lin-Wen Lee, Yi-Ning Huang, Kuang-Sheng Yeh

**Affiliations:** ^1^Department of Pediatrics, Wan Fang Hospital, Taipei Medical University, Taipei 116, Taiwan; ^2^Graduate Institute of Medical Sciences, College of Medicine, Taipei Medical University, Taipei 110, Taiwan; ^3^Department of Microbiology and Immunology, School of Medicine, College of Medicine, Taipei Medical University, Taipei 110, Taiwan; ^4^Department of Veterinary Medicine, School of Veterinary Medicine, College of Bioresources and Agriculture, National Taiwan University, Taipei 106, Taiwan

## Abstract

Static broth culture favors *Salmonella enterica* subsp. *enterica* serovar Typhimurium to produce type 1 fimbriae, while solid agar inhibits its expression. A transposon inserted in *stbC*, which would encode an usher for Stb fimbriae of a non-flagellar *Salmonella enterica* subsp. *enterica* serovar Typhimurium LB5010 strain, conferred it to agglutinate yeast cells on both cultures. RT-PCR revealed that the expression of the fimbrial subunit gene *fimA*, and *fimZ*, a regulatory gene of *fimA*, were both increased in the *stbC* mutant when grown on LB agar; *fimW*, a repressor gene of *fimA*, exhibited lower expression. Flagella were observed in the *stbC* mutant and this phenotype was correlated with the motile phenotype. Microarray data and RT-PCR indicated that the expression of three genes, *motA, motB*, and *cheM*, was enhanced in the *stbC* mutant. The *stbC* mutant was resistant to several antibiotics, consistent with the finding that expression of *yhcQ* and *ramA* was enhanced. A complementation test revealed that transforming a recombinant plasmid possessing the *stbC* restored the mannose-sensitive agglutination phenotype to the *stbC* mutant much as that in the parental *Salmonella enterica* subsp. *enterica* serovar Typhimurium LB5010 strain, indicating the possibility of an interplay of different fimbrial systems in coordinating their expression.

## 1. Introduction

*Salmonella enterica* subsp. *enterica* contains more than 2,300 serovars; one of these, Typhimurium, is an important cause of gastroenteritis [1]. The ability to adhere to the host epithelial cell is considered a prerequisite step for infection. Fimbriae, proteinaceous hair-like appendages on the outer membrane of bacteria, have been implicated in such adherence. Many types of fimbriae have been described for* Salmonella enterica* subsp. *enterica* serovar Typhimurium, among which type 1 fimbriae is the most common; therefore, type 1 fimbriae are also referred to as common fimbriae [2]. Type 1 fimbriae adhere to a variety of cells, including erythrocytes, leukocytes, intestinal cells, and even plant root hairs. More than 80% of *Salmonella *species isolates produce type 1 fimbriae, and this fimbrial type may play some role in the life cycle of bacteria [3].

 Old et al. described the phenotypic variation of the expression of type 1 fimbriae in *Salmonella enterica* subsp. *enterica* serovar Typhimurium [4, 5]. In brief, strongly type 1 fimbriate-phase bacterial cells were obtained following successive passage every 48 hr in static broth culture medium, while nonfimbriated-phase bacteria were found when cultured on solid media [4, 5]. Subsequent investigations have indicated that phenotypic expression of type 1 fimbriae in *Salmonella enterica* subsp. *enterica* serovar Typhimurium involves the interaction of several genes in the *fim* gene cluster [6–9]. To explore whether there is another genetic determinant outside the *fim* genes that would also participate in regulation of type 1 fimbriae in* Salmonella enterica* subsp. *enterica* serovar Typhimurium, Chuang et al. constructed an insertional library to screen for mutant strains that showed different type 1 fimbrial phenotypes than the parental strain [10]. Yeast agglutination test and southern blot analysis were used to screen for those mutants and validated that the transpositional event occurred only once. A group of genes classified as having fimbrial biosynthesis and regulation were involved in the expression of type 1 fimbriae in response to different culture conditions [10]. One mutant *stbC* strain agglutinated yeast obtained either from static LB broth or on solid LB agar medium, and the agglutination was mannose-sensitive [10]. The gene *stbC* encoded the usher protein for Stb fimbriae in *Salmonella enterica* subsp. *enterica* serovar Typhimurium [11]. The usher protein helps anchor the developing fimbrial subunit structure to the outer membrane [12]. Cross-talk between different fimbrial systems in one strain has been documented [13–15]. Therefore, the interaction of *fim *and *stb* fimbrial systems is an intriguing topic. This paper describes the interesting characteristics of *Salmonella enterica* subsp. *enterica* serovar Typhimurium* stbC* mutant that we discovered.

## 2. Materials and Methods

### 2.1. Bacterial Strains and Culture Media

The *Salmonella enterica* subsp. *enterica* serovar Typhimurium strain used in this study was the LT2 derivative strain LB5010 [16]. The *stbC* mutant strain was obtained from the *Salmonella enterica* subsp. *enterica* serovar Typhimurium transposon library collection in our laboratory [10]. This strain has a transposon inserted in the position of *stbC*, encoding the usher protein of StbA fimbriae. *Salmonella *cells were cultured in Luria-Bertani (LB) broth (Difco/Becton Dickinson, Franklin Lakes, NJ) or on LB agar. Mueller-Hinton agar (Difco/Becton Dickinson) was used when performing the antimicrobial susceptibility test, and modified semisolid Rappaport-Vassiliadis (MSRV) (Difco/Becton Dickinson) was used to detect the motility of *Salmonella *strains.

### 2.2. Yeast Agglutination Test

The LB agar plates were incubated at 37°C for 18 h, while the broth preparations were incubated statically at 37°C for 48 h. Bacterial cells from the solid agar were collected by a sterile loop and resuspended in 100 *μ*L of 1 × phosphate-buffered saline (PBS). Cells in the broth medium were collected by centrifugation, and the pellet was resuspended in 100 *μ*L of 1 × PBS. Subsequently, 30 *μ*L of a 3% (vol/vol) suspension of *Candida albicans* in PBS and an equal amount of bacterial cells to be tested were mixed together on a glass slide [4]. Visible agglutination after gentle agitation indicated a positive reaction for the presence of type 1 fimbriae. Any bacterial suspension that produced type 1 fimbriae was further mixed with *C. albicans,* along with 3% (wt/vol) of a D-mannose solution (Sigma Chemical Company, St. Louis, MO). The mannose-sensitive agglutination conferred by type 1 fimbriae was inhibited in the presence of mannose.

### 2.3. Electron Microscopy

Bacterial strains were grown in static broth or on solid agar and resuspended in 1 × PBS. The bacterial cells were then negatively stained with 2% phosphotungstic acid and observed with a Hitachi H-600 transmission electron microscope (Hitachi Ltd., Tokyo, Japan).

### 2.4. Motility Test

*Salmonella enterica* subsp. *enterica* serovar Typhimurium LB5010 and the *stbC* mutant strains were grown in static broth for 48 h and on solid agar for 18 h. Cells were resuspended in 1 × PBS and adjusted to the same turbidity. A drop of cell suspension was then spotted on the modified semisolid Rappaport-Vassiliadis (MSRV) agar medium and incubated at 42°C for 16 h.

### 2.5. Antimicrobial Susceptibility Test

*Salmonella enterica* subsp. *enterica* serovar Typhimurium LB5010 and the *stbC* mutant strains were analyzed for drug resistance. Antimicrobial susceptibility was tested by a disc diffusion method using commercially available discs and Mueller-Hinton agar. The following antibiotics were used: ampicillin (10 *μ*g), penicillin (10 *μ*g), cephalothin (30 *μ*g), tetracycline (30 *μ*g), doxycycline (10 *μ*g), chloramphenicol (50 *μ*g), florfenicol (30 *μ*g), streptomycin (50 *μ*g), and ciprofloxacin (5 *μ*g). *Escherichia coli* ATCC 25922 was used as the control strain. The results were interpreted according to criteria specified by the Clinical and Laboratory Standards Institute (CLSI) [17].

### 2.6. RNA Purification

Bacterial cells were harvested in TRIzole Reagent (Invitrogen, Carlsbad, CA) and disrupted using the MagNA Lyser System (Roche Diagnostics, Mannheim, Germany), with ceramic bead shaking at 5,000 rpm for 15 sec. After phenol-chloroform extraction, the aqueous layer was applied to an RNeasy column (Qiagen, Valencia, CA) for RNA purification according to the protocol provided by the manufacturer. The RNA was quantified by using a ND-1000 spectrophotometer (NanoDrop Technology, Wilmington, DE) and then examined with a Bioanalyzer 2100 (Agilent Technology, Palo Alto, CA) with an RNA 6000 Nano LabChip kit (Agilent). To enhance the sensitivity of microarray signal, the purified total RNA was subjected to a ribosomal RNA removal procedure by MICROB Express Bacterial mRNA Purification Kit (Ambion, Applied Biosystems, Foster City, CA).

### 2.7. Custom Array Design

Probes were designed by eArray of Agilent Technologies. In the design process, 4,718 *Salmonella enterica* subsp. *enterica* serovar Typhimurium LT2 transcripts were uploaded to Agilent eArray and designed by Tm matching methodology. Target sequences that were duplicated were removed. Probes with Tm at around 80°C, optimal base content, and low cross-hybridization were selected. The resulting 4,646 probes were then generated. The custom microarray was manufactured in 4 × 44 k format by in situ synthesis of oligonucleotide probes. Each array consisted of 4,646 *Salmonella enterica* subsp. *enterica* serovar Typhimurium LT2-specific probes and printed in 9 replicates.

### 2.8. Microarray Experiment

One microgram of enriched mRNA was reverse-transcribed to cDNA with a CyScribe 1st-strand cDNA labeling kit (GE Healthcare, Buckinghamshire, UK) and labeled with Cy3-CTP or Cy5-CTP (CyDye, PerkinElmer, Waltham, MA). Correspondingly labeled cDNA was then pooled and hybridized to microarrays at 60°C for 17 h. After washing and drying by nitrogen gun blowing, microarrays were scanned with an Agilent microarray scanner at 535 nm for Cy3-CTP and 625 nm for Cy5-CTP. Scanned images were then analyzed by Feature Extraction 9.5.3 software (Agilent). Image analysis and normalization software was used to quantify signal and background intensity for each feature and substantially normalized the data by rank-consistency-filtering LOWESS method.

### 2.9. Reverse Transcription Polymerase Chain Reaction (RT-PCR) Analysis

The bacteria were first stabilized by adding RNAProtect Bacteria Reagent (Qiagen) and total RNA was isolated by using an RNeasy Mini Kit (Qiagen) and RNase-free DNase I (1 unit/1 *μ*g RNA) (Promega, Madison, WI), according to the protocol provided by the manufacturer. RT-PCR was performed by a SuperScript III One-Step RT-PCR System (Invitrogen). RNA was denatured at 58°C for 5 min and followed by cDNA synthesis at 42°C for 30 min. The reaction was stopped by heating the bacteria at 94°C for 2 min. The following PCR programming was used: 35 cycles of denaturing at 94°C for 30 sec, annealing at 54°C for 30 sec, and extension at 72°C for 30 sec. An additional extension was performed at 72°C for 10 min. Primers used in the present study are listed in Table 1.

### 2.10. Complementation Test

The primer set, stbC-F and stbC-R, was used to amplify the *stbC* coding sequence from the genomic DNA of *Salmonella enterica* subsp. *enterica* serovar Typhimurium LB 5010 with the Epicentre FailSafe PCR PreMix Selection kit (Epicentre, Madison, WI). The resulting DNA fragment was cleaved with *Bam*HI and ligated into the pACYC 184 vector. *Hind*III and *Sal*I were then used to cleave a 2.9 kb DNA fragment possessing the *stbC* coding sequence from the aforementioned recombinant plasmid and ligated into the pBBR1MCS-5 vector that carries a gentamycin-resistant gene [18]. The resulting recombinant plasmid was transformed into the *stbC* mutant strain, and transformants were selected on gentamycin-containing (100 *μ*g/mL) LB agar medium. A transformant labeled *stbC* (pStbC) was selected for further study. For the control, the pBBR1MCS-5 vector was transformed into the *stbC* mutant, and a resulting transformant labeled *stbC* (vector) was selected.

## 3. Results

### 3.1. Yeast Agglutination Test

A *stbC* mutant prepared on both static LB broth and LB agar medium both exhibited agglutination when mixed with yeast cells on a glass slide. The addition of mannose inhibited agglutination, indicating that agglutination was mannose-sensitive. *Salmonella enterica* subsp. *enterica* serovar Typhimurium LB5010 from static broth agglutinated yeast cells, while that from agar medium did not. The *stbC* (pStbC) harboring the plasmid that contains the coding sequence of *stbC* exhibited the same agglutination pattern as the parental strain, while *stbC* (vector), containing the vector alone, prepared on both agar and broth medium, agglutinated yeast cells as did the *stbC* mutant strain.  Table 2 compares the yeast agglutination capabilities conferred by these strains.

### 3.2. Electron Microscopy

**Figure 1:**
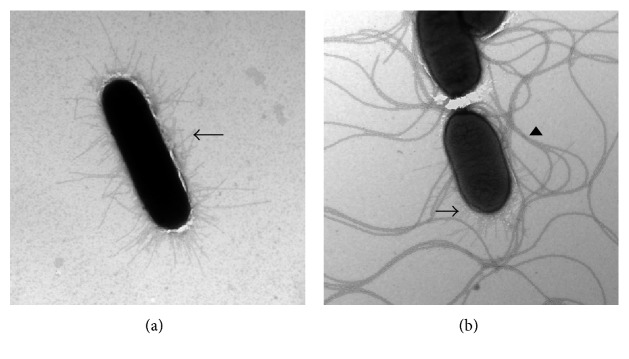
Observation of *Salmonella enterica* subsp. *enterica* serovar Typhimurium LB5010 and the *stbC* mutant strain grown in static broth culture. (a) *Salmonella enterica* subsp. *enterica* serovar Typhimurium LB5010 strain grown in static LB broth condition at 37°C for 48 h produced fimbrial appendages (arrow). No flagella structures were observed. (b) *Salmonella enterica* subsp. *enterica* serovar Typhimurium* stbC* mutant strain grown in static LB broth condition at 37°C for 48 h produced fimbrial appendages (arrow) and flagella structures (arrowhead). Bacterial cells were negatively stained with 2% of phosphotungstic acid (20,000x).

**Figure 2:**
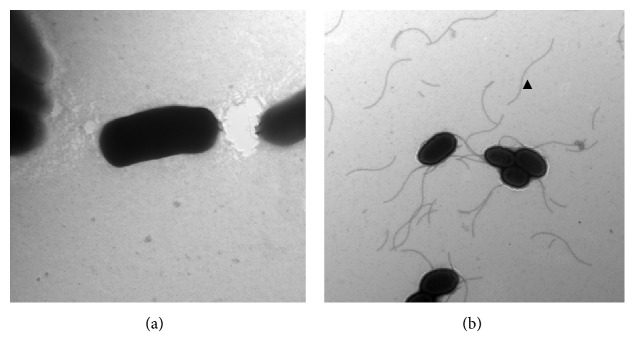
Observation of *Salmonella enterica* subsp. *enterica* serovar Typhimurium LB5010 and the *stbC* mutant strain grown on solid agar. (a) *Salmonella enterica* subsp. *enterica* serovar Typhimurium LB5010 grown on LB agar at 37°C for 16 h did not produce fimbrial appendages. (b) The *Salmonella enterica* subsp. *enterica* serovar Typhimurium* stbC* mutant grown on LB agar at 37°C for 16 h exhibited flagella structures (arrowhead) but no fimbrial appendages were observed (3,000x). Bacterial cells were negatively stained with 2% of phosphotungstic acid (20,000x).

*Salmonella enterica* subsp. *enterica* serovar Typhimurium LB5010 prepared in static LB broth culture showed fimbrial appendages on the outside of the cell (Figure 1(a)). Besides fimbriae, additional flagella-like structures were present on the *stbC* mutant strain cultured in static broth (Figure 1(b)). On the contrary, *Salmonella enterica* subsp. *enterica* serovar Typhimurium LB5010 grown on agar medium did not produce type1 fimbriae (Figure 2(a)). The *stbC* mutant prepared from agar medium did not produce fimbriae; either, however, flagella-like structures were still observed around the *stbC* mutant prepared from agar medium (Figure 2(b)).

### 3.3. Motility Test

**Figure 3:**
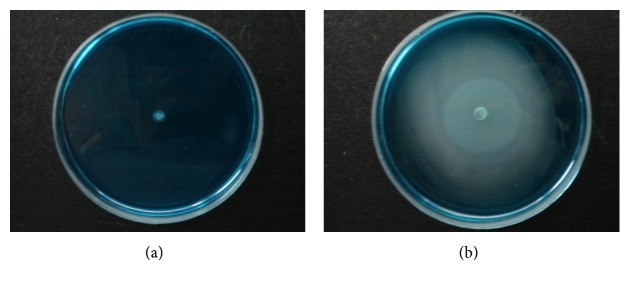
*Salmonella enterica* subsp. *enterica* serovar Typhimurium LB 5010 and the *stbC* mutant grown on MSRV agar medium. (a) *Salmonella enterica* subsp. *enterica* serovar Typhimurium LB5010 did not exhibit any “halo” appearance on the agar surface. (b) a gray-white zone was observed extending from the inoculated drop of the *stbC* mutant.

The semisolid characteristic of MSRV allows mobility to be detected as “halos” of growth around the point of inoculation. *Salmonella enterica* subsp. *enterica* serovar Typhimurium LB5010 did not exhibit a “halo” effect on the MSRV medium, and the medium remained blue around the inoculated drop (Figure 3(a)). In contrast, a gray-white zone was observed extending from the inoculated drop of the *stbC* mutant, prepared either from static LB broth or LB agar (Figure 3(b)). The* stbC* was a motile *Salmonella* strain. We also used polyvalent *Salmonella* H antiserum (Difco/Becton Dickinson) to confirm that the flagellar antigen was present on the *stbC* mutant but not on the *Salmonella enterica* subsp. *enterica* serovar Typhimurium LB5010 by slide agglutination test.

### 3.4. Antimicrobial Susceptibility Test

Since we had difficulty complementing the* stbC* with a recombinant plasmid containing the coding sequence of *stbC*, we tested whether *stbC* mutant exhibited resistance to the antibiotic marker carried in the cloning vector. We found that *stbC* was resistant to ampicillin and tetracycline carried in TA cloning and pACYC 184 vectors, respectively. Therefore, a battery of antibiotics was tested on the *stbC* mutant and the parental strain LB5010. Both strains were resistant to penicillin and streptomycin. In addition, the *stbC* mutant also exhibited resistance to ampicillin, cephalothin, tetracycline, doxycycline, chloramphenicol, florfenicol, and ciprofloxacin.

### 3.5. Microarray Analysis

Since the* stbC* mutant strain prepared from solid agar produced different results on yeast agglutination testing and exhibited multidrug resistant characteristics, it is tempting to identify the genes that would express differently between the *stbC* mutant strain and its parental strain *Salmonella enterica* subsp. *enterica* serovar Typhimurium LB5010 when grown on solid LB agar. Total RNA was isolated from both strains cultured on solid LB agar and analyzed by hybridization to a *Salmonella enterica* subsp. *enterica* serovar Typhimurium LT2 DNA microarray. The median hybridization results of 9 arrays showed that about 50 genes from the *stbC* mutant grown on agar were upregulated more than 8-fold (Table 3). Analysis of the microarray data revealed that these genes can be classified according to their functions, including fimbrial structure, motility, drug resistance, gene regulation, transportation, outer membrane porin structure, prophage, ribosomal protein, inner membrane protein, periplasmic protein, and enzymes of different functions. Twenty-two genes are enzymes with a variety of functions (44%), and these comprised the major part of the genes upregulated in the *stbC *mutant strain grown on solid agar. The detail results of the microarray assay in the present study can be accessed at GEO accession number GSE34685.

### 3.6. Reverse Transcription Polymerase Chain Reaction (RT-PCR) Analysis

To validate our microarray finding, several genes were selected and assayed by RT-PCR. The transcriptional levels of *gcvH* (5.9), *ramA* (3.9), *csgA* (2.4), *cheM* (7.2), *yhcQ *(3.0), *motA* (2.1), *motB* (3.7), STM0347 (1.5), and *rpsS* (1.4) in *stbC* were higher than those in LB5010 when both strains were grown on LB agar. (The vales in the parenthesis represent the ratio of *stbC*/LB5010.) This was correlated with the microarray data. Expression of type 1 fimbriae involved several Fim regulatory proteins and other gene products outside the *fim* gene cluster. *FimZ* and *FimY* are positive regulators for type 1 fimbriae, while *FimW* is a repressor for fimbrial expression [6–9]. 

**Figure 4:**
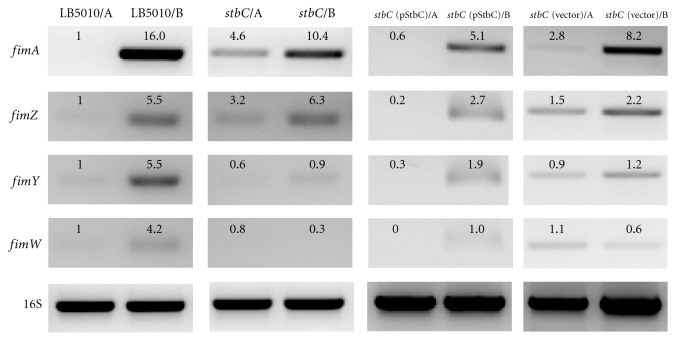
Effect of a transposon inserted in *stbC* on transcription within the *fim* gene cluster. RT-PCR assays were used to monitor *fim* gene transcription in *Salmonella enterica* subsp. *enterica* serovar Typhimurium LB5010, the *stbC* mutant, *stbC* (pStbC), and *stbC* (vector) cultured on static LB broth and solid LB agar. The intensities of the bands on the gel were determined by densitometry and are expressed relative to the value for *Salmonella enterica* subsp. *enterica* serovar Typhimurium LB5010 grown on LB agar.

 We also used RT-PCR to investigate the transcription level of the major fimbrial subunit gene *fimA* and those of three regulatory genes *fimZ*, *fimY*, and *fimW*. Total RNAs from the parental strain *Salmonella enterica* subsp. *enterica* serovar Typhimurium LB5010, *stbC* mutant, *stbC* (pStbC), and *stbC* (vector) were prepared and analyzed for *fimA*, *fimZ*,* fimY*, *fimW* mRNA, and 16S ribosomal (r)RNA expression by RT-PCR.  Figure 4 shows the RT-PCR results. Expression levels of *fim* genes of LB5010 obtained on LB agar were used as the reference. When LB 5010 was cultured in static LB broth, *fimA*,* fimZ*, *fimY*, and *fimW* had higher levels of expression than when cultured on LB agar. The *stbC* strain demonstrated the same tendency, except for *fimW*. The expression of *fimA* obtained from the* stbC* mutant strain was approximately 4.6-fold higher than that of the LB5010 strain when both strains were grown on LB agar. The expression of *fimZ* was also higher than that of LB5010 when grown on LB agar. As a control, 16S rRNA was consistently expressed in all of the strains tested. Transforming the recombinant plasmid harboring the *stbC* coding sequence to the *stbC* strain conferred on it the ability to express a level of *fimA* similar to that of LB5010. Gene expression of *fimZ*, *fimY*, and *fimW* was higher in *stbC* (StbC) grown in broth than on agar medium. The *stbC *(vector), possessing the cloning vector alone, exhibited similar gene expression patterns as the *stbC* strain.

## 4. Discussions

*Salmonella enterica *subsp. *enterica* serovar Typhimurium LB5010, a LT2 strain derivative, exhibits mannose-sensitive phenotype when grown in static broth culture but not on the solid agar medium culture. This strain is nonflagellated and not multidrug resistant. However, the present study revealed that a transposon inserted in the *stbC* gene of LB5010 strain conferred it to become a constitutively mannose-sensitive agglutinating, multidrug resistance, and flagellated strain.

Whole-genome sequence analysis of *Salmonella enterica* subsp. *enterica* serovar Typhimurium LT2 strain has revealed the presence of 13 gene clusters that contain open reading frames (ORFs) to encode putative fimbrial subunit and fimbrial-accessory proteins [11]. Laboratory-grown cultures of *Salmonella enterica* subsp. *enterica* serovar Typhimurium commonly produce only type 1 fimbriae and thin aggregative fimbriae (curli fimbriae) [19, 20]. Although the laboratory culture condition could not induce bacteria to produce Stb fimbriae, such fimbriae did express *in vivo* by flow cytometry using bovine ligated ileal loops [21]. The fact that mice infected with *Salmonella enterica* subsp. *enterica* serovar Typhimurium seroconvert to StbA, a major fimbrial subunit of Stb fimbriae, also provides evidence for *in vivo* Std fimbrial expression [21]. The amino acid sequence of the StbC subunit shares similarities to those of the usher proteins of the chaperone/usher assembly pathway. Usher protein is an integral outer membrane protein that interacts with the chaperone/fimbrial subunit complex, facilitating the release of the fimbrial subunits and their secretion through the usher channel [12, 22]. This finding leads to an interesting question: Does cross-talk occur between different fimbrial systems in one bacterial strain? For example, expression of pyelonephritis-associated pili [23] represses type 1 fimbrial expression in the same *E*. *coli* strain [14]. A transposon inserted in the *stbC* gene of *Salmonella enterica* subsp. *enterica* serovar Typhimurium conferred it to exhibit mannose-sensitive agglutination constitutively when cultured in static broth or agar medium. Nonetheless the *stbC *mutant collected from agar medium exhibited less agglutinating power than those cultured in static broth medium. Titration of the original *stbC* mutant/agar suspension to 8-fold abolished the agglutination power. One reason could be due to the decreased number of fimbriae present on the *stbC* mutant.

 Previously we have encountered difficulties when attempting to transform the recombinant plasmid containing the coding sequence of *stbC* to the* stbC* mutant to perform a complementation test. The results of our study revealed that one major reason for this could be the multidrug-resistant (MDR) phenotype of the *stbC* strain. The antimicrobial susceptibility test indicated that the *stbC* mutant resisted a battery of antibiotics. Since these antimicrobial agents have different mechanisms of antibiotic action, it is speculated that the transposon inserted in *stbC* had changed gene(s) expression that is associated with MDR. Concurrently, the microarray result indicated that *ramA* and *yhcQ* were enhanced in the *stbC *mutant compared to its parental strain LB5010. Our RT-PCR analysis also confirmed this result. RamA is a member of the AraC-XylS family of transcriptional regulators that controls one of the efflux pump genes *acrB* [24], and the product of *yhcQ* is a putative MDR pump [25]. Deletion of *yhcQ* in *E. coli* reduced the penicillin G resistance [26]. The MDR phenotype of the *stbC* mutant could be reasonably explained, at least partially, by the increased expression of *ramA *and *yhcQ*.

*Salmonella enterica* subsp. *enterica* serovar Typhimurium LB5010 was derived from LT2 strain, which is routinely used in laboratories for molecular genetics and as a representative of the wild type of *Salmonella enterica* subsp. *enterica* serovar Typhimurium [16]. This strain has defects in its flagellar synthesis gene, making it suitable for observing fimbrial appendages without flagella background [27]. Accordingly, *Salmonella enterica* subsp. *enterica* serovar Typhimurium LB5010 did not demonstrate motility on MSRV, a semisolid agar medium for isolating motile *Salmonella*. There were no flagellar structures on *Salmonella enterica* subsp. *enterica* serovar Typhimurium LB5010, prepared either from static broth or on agar medium, while the *stbC* mutant exhibited flagellar structures under electron microscopy, which was unexpected. When tested in MSRV, the *stbC* mutant exhibited extended growth from the inoculated center on MSRV, indicating the motile capability of the tested strain.

 A transposon inserted in* stbC* could cause a suppression effect that alleviated the original phenotype exhibited by LB5010. However, some flagella of the *stbC* mutant observed under electron microscopy were not anchored on the bacterial cells. This could be due to the method used to prepare the samples or to the fact that some flagella were actually secreted and without function. Interestingly, microarray data and RT-PCR also indicated that the expression of three genes, *motA*,* motB*, and *cheM,* was enhanced in the *stbC* mutant compared to the LB5010. MotA and MotB are integral to the cell membrane and are required for motor rotation [28]. The product of the *cheM* gene is a methyl-accepting chemotaxis protein II, a sensory transducer. All three genes are components of a complicated chemotaxis/flagella mechanism [29]. These results were correlated with the motile characteristic of the *stbC* mutant.

One dilemma of the *stbC* mutant strain we observed was the absence of type 1 fimbriae structures under electron microscopy. Agglutination of yeast cells did appear, and it was mannose-sensitive, indicating that the agglutination was mediated by type 1 fimbriae whose receptor contains mannose residue. RT-PCR also indicated that expression of the *fimA* was higher in the* stbC* mutant than that in the parental LB5010 when these bacteria were grown on LB agar. This finding was correlated with the fact that *stbC* had higher* fimZ* expression and lower *fimW* expression than LB5010 when grown on agar, which correlated with the previous findings. These results demonstrate that the* fimZ* gene encodes a positive regulator for *fimA*, while FimW is a repressor for *fimA* [6, 7]. FimA protein may secrete and attach on the outer membrane but did not assemble into an intact fimbrial appendage. Use of immune-gold electron microscopy would test this hypothesis. Another possibility is that* stbC* mutant grown on LB agar induced a mannose-sensitive adhesion protein that has not previously been characterized and that is present on the outer membrane. The expression of the fimbrial major subunit gene of thin aggregative fimbriae *csgA* was increased in *stbC *mutant grown on LB agar. However, this laboratory condition is not suitable for* Salmonella enterica* subsp. *enterica* serovar Typhimurium to produce thin aggregative fimbriae. Collinson et al. demonstrated that static colonization factor antigen (CFA) broth at 30°C could induce *Salmonella enterica *subsp. *enterica* serovar Enteritidis to produce thin aggregative fimbriae [30].

To better understand whether the *stbC* gene really does account for the mannose-sensitive agglutination, flagellar formation, and MDR characteristics of this interesting strain, a complementation test was performed. When a recombinant plasmid carrying the *stbC* coding sequence and a gentamycin-resistant cassette were constructed and transformed into the *stbC* strain, mannose-sensitive agglutination phenotypes and gene expression levels of the type 1 fimbrial subunit gene, *fimA*, and 3 fimbrial regulatory genes,* fimZ*,* fimY*, and *fimW*, exhibited the same tendency toward the LB5010 parental strain by the *stbC* strain. This evidence suggests that the *stbC* gene product, an usher of Stb fimbriae, may play some role in the regulatory network of type 1 fimbrial expression. The interaction of different fimbrial systems in a single strain was reported [13–15], but in the present study, we could not identify 1 or more specific *fim* genes with which StbC directly interacts, nor could we determine if other non-*fim* genes were involved in connecting these 2 different fimbrial system. However, these interesting topics warrant further investigation. Protein-protein interaction using yeast two hybrid system is being investigated in our laboratory. Complementation tests did not restore the MDR and flagellar formation characteristics of the LB5010 parental to *stbC* (pStbC). Whether this was due to polar effect or other reasons was under exploration.

## Figures and Tables

**Table 1:** Oligonucleotide primers used in the present study.

Primer	Sequence (5′-3′)	
*gvcH-RT-F*	TAAAGATCCAGCCACCG	
*gvcH-RT-R*	GGCATTACTGAACACGC	
*ramA-RT-F*	CGATTGTCGAGTGGATT	
*ramA-RT-R*	GCGTAAAGGTTTGCTGC	
*csgA-RT-F*	CGACCATTACCCAGAGC	
*csgA-RT-R*	TTGCCAAAACCAACCTG	
*cheM.-RT-F*	GCCAGATTACGCACCTC	
*cheM-RT-R*	TGCCAGCATGGAACAAC	
*yhcQ-RT-F*	ATTCCCCTGCTGCTCGT	
*yhcQ-RT-R*	ATGTCGTCGCTATTGCC	
*motA-RT-F*	GTCTGCTGCTGGTTTGG	
*motA-RT-R*	ATAGGGGCGTTCATTGT	
*motB-RT-F*	CTTTTGGCGATGTGGGT	
*motB-RT-R*	CAGCAGGGTGAAGTGGA	
*STM0347-RT-F*	GGCATCGCTTCACTCTT	
*STM0347-RT-R*	TCACCGACCGCTACATC	
*rpsS-RT-F*	ATAAGTACGAGTCGGTGCG	
*rpsS-RT-R*	CACTTGCTGAAGAAGGTAGA	
*16S-F*	TTCCTCCAGATCTCTCTACGCA	
*16S-R*	GTGGCTAATACCGCATAACG	
*fimA-RT-F*	ACTATTGCGAGTCTGATGTTTG	
*fimA-RT-R*	CGTATTTCATGATAAAGGTGGC	
*fimZ-RT-F*	ATTCGTGTGATTTGGCGT	
*fimZ-RT-R*	ACTTATCCTGTTGACCTT	
*fimY-RT-F*	GAGTTACTGAACCAACAGCT	
*fimY-RT-R*	GCCGGTAAACTACACGATGA	
*fimW-RT-F*	AAAGTGAAAGTAAAGCGG	
*fimW-RT-R*	AAGAGATAGATAATGCCG	
*stbC-F*	ATACGGGATCCCG-GCTGACAAACAGGCTGGTGATAAACAAT 3′	The underlined sequence denotes the *Bam*HI restriction site
*stbC-R*	CTACGGGATCCCG-TGACGGGCTAGGTAAACCTGATAATCTG 3	The underlined sequence denotes the *Bam*HI restriction site

**Table 2:** Yeast agglutination test of *Salmonella enterica* subsp. *enterica* serovar Typhimurium LB5010 and *stbC* mutant strains.

	Agglutination of yeast cells mixed with different concentrations of bacterial cells with a 2-fold dilution^a^					
Strain	1×	2×	4×	8×	16×	32×
LB5010/agar	−	−	−	−	−	−
LB5010/broth	+++	+++	++	+	+	−
*stbC* mutant/agar	++	++	+	−	−	−
*stbC* mutant/broth	+++	+++	+++	++	++	+
*stbC* (pStbC)/agar	−	−	−	−	−	−
*stbC* (pStbC)/broth	+++	++	++	+	+	−
*stbC* (vector)/agar	++	++	+	−	−	−
*stbC* (vector)/broth	+++	+++	+++	++	++	+

^a^Strong agglutination is indicated by (+++) and a negative result by (−).

**Table 3:** Identification of selected genes of *Salmonella enterica* subsp. *enterica* serovar Typhimurium* stbC *mutant strain grown on LB agar by microarray analysis.

Group	Function	Ratio of expression in *stbC*/Lb5010 on agar
Fimbriae		
* csgA*	Curlin major subunit	24.3
Motility		
* cheM*	Methyl-accepting sensory transducer	19.7
* motA*	Proton conductor component of motor	10.3
* motB*	Enables flagellar motor rotation	8.8
Drug resistance		
* yhcQ*	Putative membrane located multidrug resistance protein	16.9
* ramA*	Putative regulatory protein of efflux pump	33.1
Porin		
STM0346	Homologue of Ail and OmpX, putative outer membrane protein	18.4
* nmpC*	Outer membrane protein, porin	8.9
* cirA*	Pori, receptor for colicin, requires TonB	8.3
Prophage		
STM2706	Fels-2 prophage	77.7
STM2595	Gifsy-1 prophage	35.8
Gene regulation		
STM0347	LuxR family putative response regulator	16.7
Ribosomal protein		
* rpmC*	50S ribosomal subunit protein L29	8.5
* rplD*	50S ribosomal subunit protein L4	8.4
* rpsS*	30S ribosomal subunit protein S19	8.3
Transportation		
* ynfM*	Putative MFS family transport protein	32.7
* cysU*	ABC superfamily thiosulfate transport protein	11.7
* sbp*	ABC superfamily sulfate transport protein	8.9
Inner membrane protein		
STM3350	Putative inner membrane protein	11.3
* yjcB*	Putative inner membrane protein	9.7
Periplasmic protein		
STM3650	Putative periplasmic protein	10.9
* ybfA*	Putative periplasmic protein	33.1
Enzymes		
* gcvH*	Glycine cleavage complex protein H	93.7
* gcvT*	Glycine cleavage complex protein H	77.7
* aceK*	Isocitrate dehydrogenase kinase/phosphatase	27.7
* prpE*	Putative acetyl-CoA synthetase	9.6
